# Supplementary material for: The Legacy of the Idrija Mine Twenty-Five Years after Closing: Is Mercury in the Water Column of the Gulf of Trieste Still an Environmental Issue?
Source: Int J Environ Res Public Health. 2021 Sep 28;18(19):10192. doi: 10.3390/ijerph181910192 (PMC8508114; doi:10.3390/ijerph181910192)
Supplement: Supplementary file 1 [file ijerph-18-10192-s001.zip › ijerph-1348146-supplementary_clean.pdf]

Supplementary Material: Tables

Table S1. Mercury concentrations in the SPM (PHg, ng L^{-1} and µg g^{-1}) and in the dissolved fraction (DHg, ng L^{-1}) in the surface and bottom water samples collected at different sites in the vicinity of the main access channel to the Port of Monfalcone under different environmental conditions.

Sampling campaign			P1		P2		P3		P4		P5		P6	
			S	B	S	B	S	B	S	B	S	B	S	B
**1** 1 Feb	DHg	ng L^{-1}	46.5	20.9	20.3	20.5	22.5	21.3	24.0	20.5	21.3	112	21.0	20.1
	PHg	ng L^{-1}	4.49	4.45	1.69	3.96	3.71	2.10	4.02	2.98	1.52	1.81	1.55	1.45
	PHg	µg g^{-1}	1.30	1.05	0.53	1.01	0.98	0.51	1.07	0.68	0.47	0.55	0.55	0.39
	SPM	mg L^{-1}	3.55	4.27	3.20	3.91	3.78	4.06	3.75	4.40	3.25	3.32	2.92	3.64
**2** 2 Mar	DHg	ng L^{-1}	46.8	68.7	16.9	149	16.8	20.7	1.16	1.77	14.6	<LOD	2.64	<LOD
	PHg	ng L^{-1}	7.32	7.68	8.11	5.01	12.5	8.64	7.98	5.86	7.88	5.98	6.44	4.36
	PHg	µg g^{-1}	0.38	1.47	0.74	1.13	1.52	1.87	1.38	1.51	1.54	1.43	1.55	1.13
	SPM	mg L^{-1}	19.1	5.32	10.9	4.45	8.44	4.62	5.80	3.89	5.12	4.17	4.17	3.88
**3** 31 Mar	DHg	ng L^{-1}	1.86	<LOD	<LOD	<LOD	<LOD	<LOD	<LOD	<LOD	1.25	<LOD	<LOD	<LOD
	PHg	ng L^{-1}	1.01	5.69	0.65	0.61	1.00	5.70	2.15	3.58	3.05	0.70	0.66	0.59
	PHg	µg g^{-1}	0.18	0.50	0.17	0.14	0.29	1.21	0.80	0.57	1.28	0.15	0.22	0.12
	SPM	mg L^{-1}	5.79	11.4	3.93	4.23	3.45	4.75	2.82	6.24	2.51	4.61	3.04	4.87
**4** 21 Apr	DHg	ng L^{-1}	4.06	3.12	3.75	1.31	6.11	3.83	4.14	1.45	7.28	20.0	3.13	1.50
	PHg	ng L^{-1}	0.92	2.46	2.43	3.07	1.16	3.11	5.66	5.58	1.75	1.03	1.61	9.03
	PHg	µg g^{-1}	0.31	0.39	0.51	0.52	0.24	0.58	1.38	1.10	0.56	0.26	0.45	1.37
	SPM	mg L^{-1}	3.03	6.27	4.73	5.78	4.82	5.30	3.98	5.06	3.15	3.97	3.59	6.64
**5** 4 May	DHg	ng L^{-1}	4.02	6.17	3.36	5.24	<LOD	3.68	6.80	1.16	1.90	3.91	8.91	2.16
	PHg	ng L^{-1}	2.07	2.45	1.76	3.22	0.85	3.73	0.94	0.53	0.39	1.69	0.66	2.89
	PHg	µg g^{-1}	0.52	0.45	0.39	0.56	0.20	0.61	0.21	0.11	0.10	0.34	0.19	0.54
	SPM	mg L^{-1}	3.97	5.45	4.57	5.61	4.21	6.11	4.57	4.87	3.90	4.86	3.64	5.37

S: surface; B: bottom

Supplementary Material: Figures

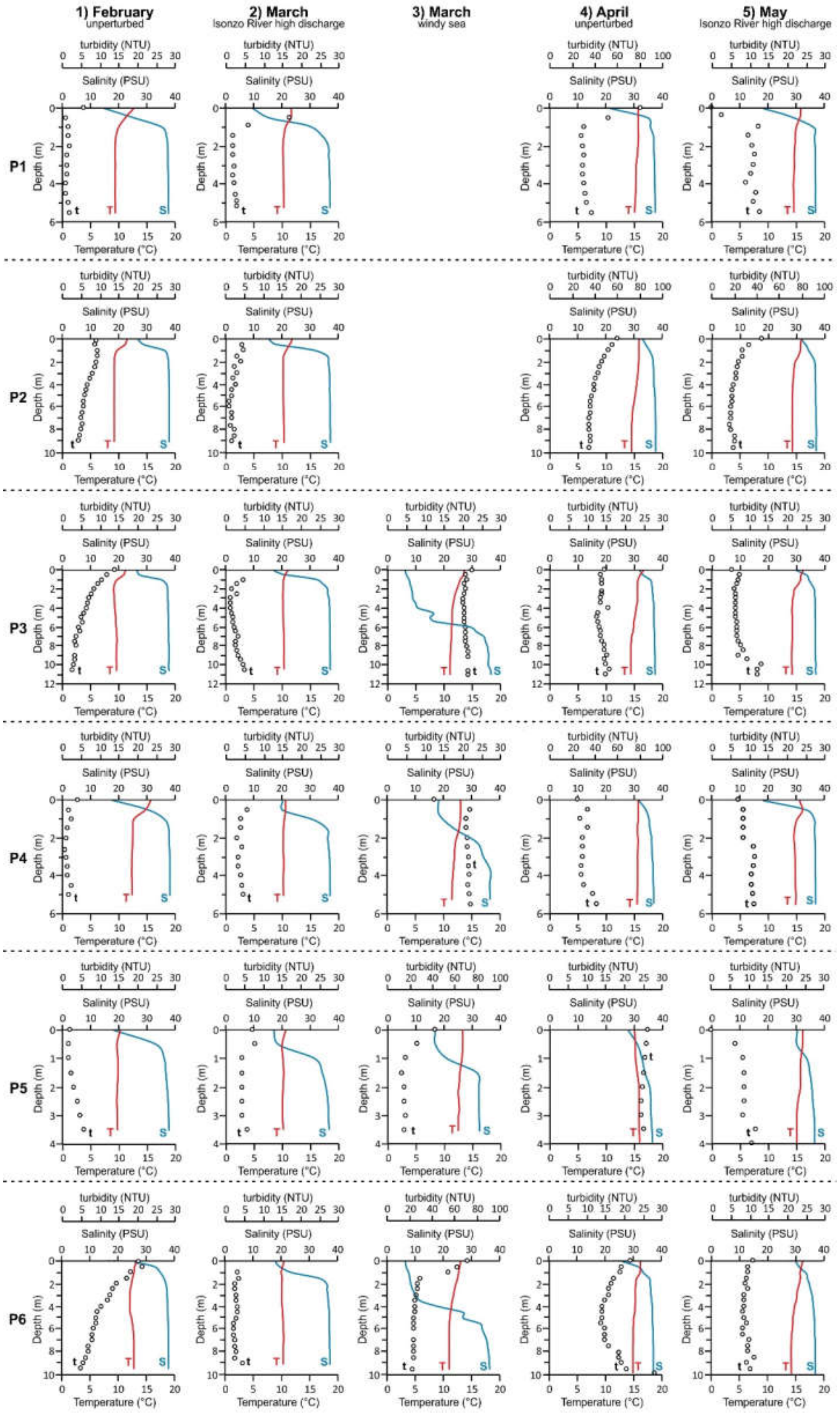

Figure S1. Vertical profiles of turbidity (NTU, grey dots), salinity (PSU, blue line) and temperature (°C, red line) recorded at the six investigated sites in the vicinity of the main access channel to the Port of Monfalcone (Bay of Panzano, Gulf of Trieste).

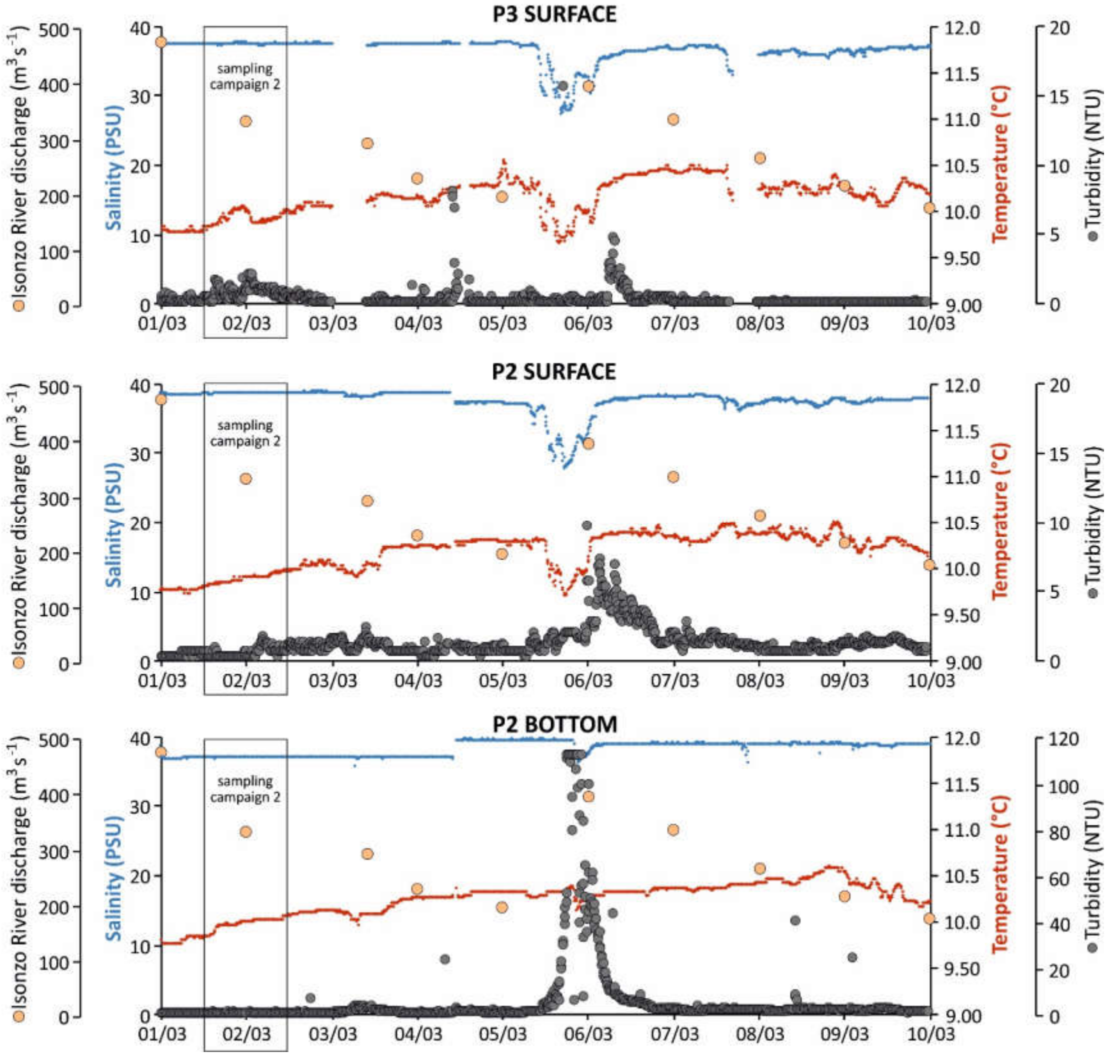

Figure S2. Isonzo River daily discharge (m^3 s^{-1}) and variations of salinity (PSU), temperature (°C) and turbidity (NTU) at site P2 (surface and bottom water layers) and site P3 (surface water layer) between the 1st and 10th March 2016.
